# Evaluating Lignocellulosic Biomass, Its Derivatives, and Downstream Products with Raman Spectroscopy

**DOI:** 10.3389/fbioe.2015.00050

**Published:** 2015-04-20

**Authors:** Jason S. Lupoi, Erica Gjersing, Mark F. Davis

**Affiliations:** ^1^Oak Ridge National Laboratory, BioEnergy Science Center, Oak Ridge, TN, USA; ^2^National Renewable Energy Laboratory, National Bioenergy Center, Golden, CO, USA

**Keywords:** Raman spectroscopy, high-throughput, lignin, glucose, xylose, process monitoring, cellulose, ethanol

## Abstract

The creation of fuels, chemicals, and materials from plants can aid in replacing products fabricated from non-renewable energy sources. Before using biomass in downstream applications, it must be characterized to assess chemical traits, such as cellulose, lignin, or lignin monomer content, or the sugars released following an acid or enzymatic hydrolysis. The measurement of these traits allows researchers to gage the recalcitrance of the plants and develop efficient deconstruction strategies to maximize yields. Standard methods for assessing biomass phenotypes often have experimental protocols that limit their use for screening sizeable numbers of plant species. Raman spectroscopy, a non-destructive, non-invasive vibrational spectroscopy technique, is capable of providing qualitative, structural information and quantitative measurements. Applications of Raman spectroscopy have aided in alleviating the constraints of standard methods by coupling spectral data with multivariate analysis to construct models capable of predicting analytes. Hydrolysis and fermentation products, such as glucose and ethanol, can be quantified off-, at-, or on-line. Raman imaging has enabled researchers to develop a visual understanding of reactions, such as different pretreatment strategies, in real-time, while also providing integral chemical information. This review provides an overview of what Raman spectroscopy is, and how it has been applied to the analysis of whole lignocellulosic biomass, its derivatives, and downstream process monitoring.

## Introduction

The production of fuels, chemicals, and materials from plants has offered an opportunity to supplant usage of products fashioned from non-renewable energy sources. Lignocellulosic biomass is predominantly composed of cellulose, non-cellulosic polysaccharides (NCPs), and lignin, and provides a useful starting feedstock for industrial processes. Before a specific plant can be considered for downstream applications, the chemical traits of the biomass must be characterized. These assessments include, but are not limited to, the compositional analysis of the plant’s cellulose, NCP, and lignin contents, the ratio of syringyl (S), guaiacyl (G), and *p*-hydroxyphenol (H) lignin monomers, the release of simple sugars following an acid or enzymatic hydrolysis, and the cellulose crystallinity index. Many of these evaluations gage the recalcitrance of the plant cell wall, and enable researchers to develop appropriate pretreatment strategies to deconstruct the biomass (Blanch et al., 2011), or genetic strategies to synthesize a more ideal starting feedstock (Furtado et al., 2014). The standard methods developed for biomass characterization are beneficial for evaluating small sample sets, but specific experimental attributes limit their use for screening large arrays of prospective plants to isolate those possessing quintessential traits for biofuel and/or biomaterial production. These attributes include laborious sample preparation protocols [derivatization of samples in gas chromatography (GC) analysis and sample clean-up for liquid chromatography or GC], use of toxic reagents that may require remediation (acetyl bromide, boron trifluoride etherate, trifluoroacetic acid, sulfuric acid), long analysis times [chromatography, nuclear magnetic resonance (NMR)], complex data analysis [pyrolysis GC/mass spectrometry (MS) analysis of lignin monomer content], and/or the destruction of the sample (pyrolysis, GC, solution state NMR). In order to circumvent some of these limitations, techniques have been developed that are non-destructive, require little to no sample preparation, and have increased throughout, allowing more plants to be assessed in less time and with reduced experimental costs (Lupoi et al., 2014b).

**Figure 1:**
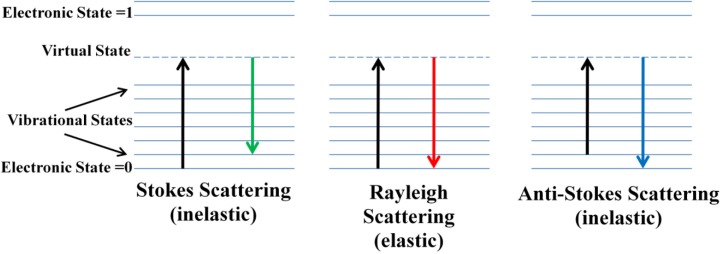
**Energy diagrams of Stokes, Rayleigh, and anti-Stokes Raman scattering**. Photons from the excitation source collide with the molecule, promoting the molecule to a short-lived virtual state, whereby it instantaneously emits energy and relaxes back to the lowest vibrational state with identical frequency to the incident light (Rayleigh), has a net increase in energy (Stokes), or has lost energy (anti-Stokes) [from Lupoi, “Developments in enzyme immobilization and near-infrared Raman spectroscopy with downstream renewable energy applications” (2012). *Graduate Theses and Dissertations*. Paper 12732.].

The phenomenon of Raman scatter was first envisaged theoretically in 1921 by Smekal, and was proven experimentally in 1928 by Raman and Krishnan, as well as Lansberg and Mandelstam (Smekal, 1923; Landsberg and Mandelstam, 1928; Raman and Krishnan, 1928). Raman spectroscopy is a vibrational spectroscopy technique in which the scattered photons, generated during the interaction between light and matter, are measured. While the light source C. V. Raman used was sunlight, modern applications of Raman spectroscopy employ ultraviolet (UV), visible, or near-infrared (NIR) lasers. The scattering produced can have an identical (elastic), higher (inelastic), or lower (inelastic) frequency than that of the excitation source [Figure 1; Lupoi (2012)]. These types of scattering are named Rayleigh, Stokes, and anti-Stokes, respectively (Carey, 1982; McCreery, 2000; Smith and Dent, 2005; Popp, 2006). Rayleigh scattering is the most intense, and needs to be thoroughly removed from the optical beam path using specialized optics such as holographic notch filters (HNFs) (Smith and Dent, 2005; Dao, 2006). If not eliminated, Rayleigh scattering can lead to saturation of the detector, and can obscure Raman signal from Stokes scattering, a much weaker phenomenon, as only approximately one per one million photons generated lead to this type of inelastic scattering (Smith and Dent, 2005). Stokes scattering is the most common type measured using Raman spectroscopy, and results in an energy shift to higher vibrational levels. Anti-Stokes scattering results in a shift from a higher to lower vibrational levels, and is less common due to the lower probability of molecules populating higher vibrational levels at ambient conditions. An important feature of the Raman phenomenon is that, unlike in infrared (IR) spectroscopy, molecules are promoted to short-lived, virtual vibrational levels (Figure 1). Therefore, matching the excitation frequency to that necessary to promote molecules from the ground state to the first excited vibrational level is not requisite.

A molecule is considered “Raman active” if there is a change in the polarizability of the electron cloud during the interaction of the molecule with light. Vibrational modes including C–C, C = C, C–H, C–O, H–C–C, C–O–H, H–C–H, etc., can be expected in an archetypal Raman spectrum (Wiley and Atalla, 1987; Agarwal and Ralph, 1997; Larsen and Barsberg, 2010; Agarwal et al., 2011). As a rule of thumb, symmetric bonds will have the largest changes in polarizability and therefore the strongest Raman signals. Table 1 lists representative vibrational modes measured in biomass constituents, and their respective band assignments. In contrast to Raman, a change in dipole moment leads to molecules being “IR active” in IR spectroscopy. Therefore, asymmetric bonds have strong peaks in IR spectra. This difference in selection rules signifies that these two techniques provide complementary information. Vibrational modes that are Raman active will not be present or have small contributions in IR spectra, and vice versa. If a molecule has a center of symmetry, the principle of mutual exclusion states that the molecule will either be IR *or* Raman active. Some non-centrosymmetric molecules, such as those possessing C_1_ symmetry, and hence no symmetry, can have both IR and Raman active vibrational modes (Ingle and Crouch, 1988). Examples of these types of molecules include isopropyl alcohol, propylene glycol, and 2-butanol (National Institute of Standards and Technology (NIST), 2013). The diatomic nitric oxide is another molecule that, although it produces only one peak, gives rise to IR and Raman active modes, since there is both a change in dipole and polarizability (Smith and Dent, 2005). Another significant difference between the two techniques is the ability of Raman spectroscopy to be used for measuring aqueous and biological samples, whereas IR spectra are appreciably hindered by the presence of water. Lastly, Raman spectra are often less complex than IR spectra due to the diminished signals of overtone and combination vibrational modes, leading to more spectrally resolved peaks.

**Table 1:** **Vibrational modes and band assignments measured in lignocellulosic biomass**.

Vibrational mode cm^−1^	Biomass constituent	Assignment
311	Cellulose	CCO torsion (Schenzel and Fischer, 2001)
329	Cellulose	CCC, CO, CCO, ring deformation (Schenzel and Fischer, 2001)
331	Cellulose	Heavy atom bending (Wiley and Atalla, 1987)
337	Lignin	Ring deformation, OH torsion (Larsen and Barsberg, 2010)
344	Cellulose	Heavy atom stretching (Wiley and Atalla, 1987), CCC, CO, CCO, ring deformation (Schenzel and Fischer, 2001)
352	Cellulose	CCC, CO, CCO, ring deformation; cellulose II (Schenzel and Fischer, 2001)
357	Lignin	Aromatic ring substituents torsion (Larsen and Barsberg, 2010)
369	Lignin	Skeletal deformation (Agarwal and Atalla, 2010); COC symmetric, in-plane bend (Larsen and Barsberg, 2010)
373	Cellulose	CCC, CO, CCO, ring deformation; cellulose II (Schenzel and Fischer, 2001)
380	Cellulose	Heavy atom stretching (Wiley and Atalla, 1987), CCC, CO, CCO, ring deformation (Schenzel and Fischer, 2001)
418	Cellulose	CCC, CCO, ring deformation; cellulose II (Schenzel and Fischer, 2001)
437	Cellulose	Heavy atom stretching (Wiley and Atalla, 1987), CCC, CCO, ring deformation (Schenzel and Fischer, 2001)
458	Cellulose	Heavy atom stretching (Wiley and Atalla, 1987); CCC, CCO, ring deformation (Schenzel and Fischer, 2001)
468	Lignin	Ring deformation (Larsen and Barsberg, 2010)
503	NCSPs	CCO (Kacurikova et al., 1998)
519	Cellulose	Heavy atom stretching (Wiley and Atalla, 1987)
521	NCSPs	CCO (Kacurikova et al., 1998)
531	Lignin	Skeletal deformation (Agarwal and Atalla, 2010)
	NCSPs	CCO (Kacurikova et al., 1998)
553	NCSPs	CCO (Kacurikova et al., 1998)
559	Lignin	CCO and CCC in-plane bend (Larsen and Barsberg, 2010)
582	Lignin	Ring deformation (Larsen and Barsberg, 2010)
597	Lignin	Skeletal deformation (Agarwal and Atalla, 2010)
609	Cellulose	CCH torsion (Schenzel and Fischer, 2001)
637–644	Lignin	Ring and skeletal deformation (Agarwal, 1999; Larsen and Barsberg, 2010)
645	β-Pinene	Ring deformation (Schulz and Baranska, 2007)
652	1,8-Cineol (eucalyptol)	Ring deformation (Schulz and Baranska, 2007)
666	α-Pinene	Ring deformation (Schulz and Baranska, 2007)
712	Lignin	Ring deformation; CC stretch (Larsen and Barsberg, 2010)
780	Lignin	CO stretch (Larsen and Barsberg, 2010); lignin aromatic skeletal vibrations (Agarwal, 1999; Larsen and Barsberg, 2010)
793	Lignin	Out-of-plane CH bend; ring deformation; CO stretch (Larsen and Barsberg, 2010)
799	Lignin	Ring deformation; CO stretch (Larsen and Barsberg, 2010)
805	Lignin	CO stretch; aryl symmetric CH bend; CH out-of-plane bend (Larsen and Barsberg, 2010)
829	Lignin	CH out-of-plane bend (Larsen and Barsberg, 2010)
843	Lignin	Breathing mode (Larsen and Barsberg, 2010)
854	Pectin	(C–O–C) Skeletal mode of α-anomers (Schulz and Baranska, 2007)
885	Cellobiose	(C–O–C) Skeletal mode (Schulz and Baranska, 2007)
897	Cellulose, NCSPs	CH, ring (Kacurikova et al., 1998), C1–H (Schenzel and Fischer, 2001)
904	Cellulose, NCSPs	CH, ring (Kacurikova et al., 1998; Schenzel and Fischer, 2001)
910	Cellulose	HCC and HCO bending (Wiley and Atalla, 1987)
920–932	Lignin	CCH wag (Agarwal, 1999)
921	Lignin	Ring deformation; in-plane CC stretch; COC stretch (Larsen and Barsberg, 2010)
942	Lignin	Lignin CCH wag; aromatic skeletal vibrations (Larsen and Barsberg, 2010; Agarwal et al., 2011)
969	Cellulose	CC and CO stretching (Wiley and Atalla, 1987), CH_2_ (Schenzel and Fischer, 2001)
977	Lignin	Lignin CCH and –HC = CH– deformation; methyl wagging (Larsen and Barsberg, 2010; Agarwal et al., 2011)
	Cellulose	CO (Kacurikova et al., 1998)
995	Cellulose	CC and CO stretching (Wiley and Atalla, 1987)
997	Cellulose	CH_2_ (Schenzel and Fischer, 2001)
1000–1010	Cellulose, NCSPs	CC and COH (Kacurikova et al., 1998)
1026	NCSPs	CC and COH (Kacurikova et al., 1998)
1033	Lignin	Lignin CH_3_ wagging; CH_3_ out-of-plane rock; aromatic skeletal vibrations, methoxy vibrations (Larsen and Barsberg, 2010; Agarwal et al., 2011)
1037	Cellulose	CC and CO stretching (Wiley and Atalla, 1987)
1043	Lignin	OC stretch; ring deformation; CH_3_ wagging (Larsen and Barsberg, 2010)
1057	Cellulose	CC and CO stretching (Wiley and Atalla, 1987)
1089	NCSPs	COC, C–C, ring vibration (Kacurikova et al., 1998)
1095	Cellulose	CC and CO stretching (Wiley and Atalla, 1987), COC, glycosidic; ring breathing, symmetric (Schenzel and Fischer, 2001)
1101	NCSPs	COC, C–C, ring vibration (Kacurikova et al., 1998)
1106	NCSPs	COC, C–C, ring vibration (Kacurikova et al., 1998)
1115	Cellulose	COC, glycosidic; ring breathing, symmetric; cellulose II (Schenzel and Fischer, 2001)
1117	Lignin	Lignin methoxy vibrations; aryl CH bend (Larsen and Barsberg, 2010)
	Cellulose	CC and CO stretching (Wiley and Atalla, 1987)
1121	Cellulose	CC and CO stretching (Wiley and Atalla, 1987), COC, glycosidic; ring breathing, symmetric (Schenzel and Fischer, 2001)
1125	Cellulose, NCSPs	COC and C–C (Kacurikova et al., 1998)
1130–1136	Lignin	Coniferaldehyde/sinapaldehyde mode (Agarwal and Atalla, 2010)
1142	Cellulose	CC, CO ring breathing, asymmetric; cellulose II (Schenzel and Fischer, 2001)
1147	Lignin	Lignin methoxy vibrations; aromatic CCH bend (Larsen and Barsberg, 2010)
1151	Cellulose	Stretching and HCC and HCO bending (Wiley and Atalla, 1987), CC, CO ring breathing, asymmetric (Schenzel and Fischer, 2001)
1155	Carotenoids	C–C stretch (Schulz and Baranska, 2007)
1169	Lignin	Lignin hydroxyl COH bend; aromatic skeletal vibrations (Larsen and Barsberg, 2010)
1185	Lignin	Lignin methoxy vibrations; COH in-plane bend (Larsen and Barsberg, 2010)
1199	Lignin	In-plane CH stretch (Larsen and Barsberg, 2010)
1202	Lignin	Lignin methoxy vibrations (Larsen and Barsberg, 2010)
	Cellulose	CH_2_, HCC, HOC, COH (Schenzel and Fischer, 2001)
1214–1217	Lignin	Aryl-O of aryl OH and aryl-OCH_3_; ring deformation (Agarwal, 1999; Larsen and Barsberg, 2010)
1256	Lignin	CO stretch (Larsen and Barsberg, 2010)
1262	Cellulose	CH_2_, HCC, HOC, COH (Schenzel and Fischer, 2001)
1268	Lignin	Lignin aromatic skeletal vibrations; methoxy vibrations (Agarwal, 1999; Agarwal et al., 2011)
1272	Lignin	Ring deformation; CO stretch (Larsen and Barsberg, 2010)
1275	Cellulose	HCC and HCO bending (Wiley and Atalla, 1987)
1288	Lignin	Ring deformation and in-plane CH and COH bend (Larsen and Barsberg, 2010)
1291	Cellulose	HCC and HCO bending (Wiley and Atalla, 1987), CH_2_, HCC, HCO, COH (Schenzel and Fischer, 2001)
1298	Lignin	CH, CC stretch; ring deformation (Larsen and Barsberg, 2010)
1312	NCSPs	CH, COH (Kacurikova et al., 1998)
1331	Lignin	Aliphatic OH stretch (Agarwal and Atalla, 2010)
	Cellulose	HCC and HCO bending (Wiley and Atalla, 1987)
1337	Cellulose	HCC, HCO, and HOC bending (Wiley and Atalla, 1987), CH_2_, HCC, HCO, COH (Schenzel and Fischer, 2001)
1365	NCSPs	CH, OH stretch (Kacurikova et al., 1998)
1373	NCSPs	CH_2_, HCC, HCO, COH; cellulose II (Schenzel and Fischer, 2001)
1372–1383	Lignin	Phenolic OH stretch
1376	NCSPs	CH, OH stretch (Kacurikova et al., 1998)
1378	Cellulose	HCC, HCO, and HOC bending (Wiley and Atalla, 1987; Schenzel and Fischer, 2001), CH_2_ (Schenzel and Fischer, 2001)
1378–1390	Lignin	Phenolic OH (Agarwal, 1999)
1380	Lignin	Umbrella CH bend (Larsen and Barsberg, 2010)
1407	Cellulose	HCC, HCO, and HOC bending (Wiley and Atalla, 1987; Schenzel and Fischer, 2001), CH_2_ (Schenzel and Fischer, 2001)
1427	Lignin	Lignin methoxy deformation, methyl bending, aromatic skeletal vibrations (Agarwal, 1999; Agarwal et al., 2011)
1455	Lignin	CH_3_ scissoring; CH_3_ out-of-plane bend; umbrella bend (Larsen and Barsberg, 2010)
1456	Cellulose	HCH and HOC bending (Wiley and Atalla, 1987; Schenzel and Fischer, 2001), CH_2_ scissoring (Schenzel and Fischer, 2001)
1460	Lignin	Lignin methoxy deformation, CH_2_ scissoring (Agarwal and Atalla, 2010)
1461	Cellulose	CH_2_ scissoring (Schenzel and Fischer, 2001)
1465	Lignin	CH_3_ scissoring; CH_3_ out-of-phase bend (Larsen and Barsberg, 2010)
1475	Cellulose	HCH and HOC bending (Wiley and Atalla, 1987), CH_2_ scissoring (Schenzel and Fischer, 2001)
1506–1514	Lignin	Aryl ring stretch, asymmetric (Agarwal, 1999)
1517–1521	Lignin	Asymmetric aryl ring stretch (Agarwal, 1999)
1528	Carotenoids	C = C stretch (Schulz and Baranska, 2007)
1605	Lignin	Lignin aromatic skeletal vibrations (Agarwal, 1999; Larsen and Barsberg, 2010)
1632	Lignin	Lignin C = C stretch of coniferaldehyde, sinapaldehyde, phenolic esters (Agarwal, 1999; Agarwal et al., 2011)
1643	α-Pinene	C = C stretch (Schulz and Baranska, 2007)
1656	Lignin	Lignin C = C stretch of coniferyl alcohol and sinapyl alcohol (Agarwal, 1999; Agarwal et al., 2011)
1659	β-Pinene	C = C stretch (Schulz and Baranska, 2007)
1704	Lignin	Carbonyl stretch
1739	NCSPs	C = O stretch (Schulz and Baranska, 2007)
1745	Pectin	C = O stretch (Schulz and Baranska, 2007)
2842	Lignin	Out-of-plane CH symmetric stretch (Larsen and Barsberg, 2010)
2845	Lignin	CH stretch in OCH_3_, symmetric (Agarwal and Atalla, 2010)
2853	Cellulose	CH_2_ symmetric stretch (Liang and Marchessault, 1959)
2859	Lignin	Out-of-plane symmetric CH stretch (Larsen and Barsberg, 2010)
2866	Cellulose	CH and CH_2_ stretching (Wiley and Atalla, 1987)
2867	Lignin	Out-of-plane symmetric CH stretch (Larsen and Barsberg, 2010)
2889	Cellulose	CH and CH_2_ stretching (Wiley and Atalla, 1987)
2917	NCSPs	CH stretch (Kacurikova et al., 1998)
2922	Lignin	Out-of-plane asymmetric CH stretch (Larsen and Barsberg, 2010)
2933	NCSPs	CH stretch (Kacurikova et al., 1998)
2939	Lignin	CH stretch in OCH_3_, asymmetric (Agarwal and Atalla, 2010; Larsen and Barsberg, 2010)
2943	Cellulose	CH and CH_2_ stretching (Wiley and Atalla, 1987)
2963	Cellulose	CH and CH_2_ stretching (Wiley and Atalla, 1987)
3005	Lignin	In-plane CH stretch (Larsen and Barsberg, 2010)
3014	Lignin	In-plane CH stretch (Larsen and Barsberg, 2010)
3039	Lignin	In-plane CH stretch (Larsen and Barsberg, 2010)
3062	Lignin	In-plane CH stretch (Larsen and Barsberg, 2010)
3071	Lignin	Aromatic CH (Agarwal and Atalla, 2010)
3286	Cellulose	OH stretch (Wiley and Atalla, 1987)
3335	Cellulose	OH stretch (Wiley and Atalla, 1987)
3363	Cellulose	OH stretch (Wiley and Atalla, 1987)
3402	Cellulose	OH stretch (Wiley and Atalla, 1987)

*NCSPs, non-cellulosic structural polysaccharides*.

Typical Raman instruments are composed of the excitation source (i.e., lasers), beam-steering optics to focus the incident light onto the sample, collect the generated scattering, and guide the scattering to the entrance slit of a spectrometer (McCreery, 2000; Dao, 2006; Meyer et al., 2011). HNFs block Rayleigh scattering from entering the spectrometer. Dispersive instruments utilize gratings to diffract the scattering. The diffracted light is projected through the exit slit, and onto a detector. Various detectors, such as the charge-couple device or indium gallium arsenide NIR detector convert light into an electronic response, producing the Raman spectrum. The selection of the excitation wavelength is critical to obtaining quality Raman spectra, as the Raman intensity is directly proportional to the incident frequency to the fourth power. Therefore, when employing UV or visible lasers, the Raman signal, in theory, will be more intense. This is not often achieved in practice when using visible excitation to measure biomass, as the intrinsic fluorescence generated from plants can significantly conceal the Raman signal. Higher energy UV lasers require some precautions to be taken, such as the prevention of sample degradation, which can be achieved by equipping the instrument with a rotating stage, and the enactment of adequate safety strategies. Researchers have developed various techniques to combat the difficulty in obtaining Raman spectra from highly fluorescent molecules like lignin. These methods have typically included the employment of NIR excitation sources, such as 1064 nm neodymium-doped yttrium orthovanadate or neodymium-doped yttrium aluminum garnet lasers (Agarwal et al., 2011; Meyer et al., 2011; Lupoi and Smith, 2012). NIR lasers, having the longest wavelength, lead to diminished spectral intensities. Conversely, since fluorescence emission maxima occur at lower wavelength regions, the employment of NIR excitation can result in significantly reduced spectral background. As an example, the use of a 785 nm laser, juxtaposed to a 1064 nm laser, will produce 3.8-times more scattering (Meyer et al., 2011). The analysis of a lignin sample using both excitations, however, revealed a background that was 160-times higher when employing the higher frequency 785 nm laser (Meyer et al., 2011). Most of these applications utilizing NIR lasers have been Fourier-transform Raman (FT-Raman) spectroscopy experiments. However, instrumental advances, such as better detectors for NIR wavelengths, have enabled NIR, dispersive Raman spectroscopy to provide a lower cost alternative to FT-Raman systems (Chase and Talmi, 1991; Lewis et al., 1993; Barbillat and Da Silva, 1997). Other instrumental methods like coherent anti-Stokes Raman scattering (CARS) and stimulated Raman scattering (SRS) spectroscopies have also provided fluorescence free Raman spectra (Saar et al., 2010; Zeng et al., 2012; Pohling et al., 2014).

Due to the complex composition of biomass, Raman spectra should be prudently interpreted. There can be significant spectral overlap between vibrational modes, challenging a routine spectral assignment of peaks. Cellulose and hemicellulose are structurally similar, and therefore, exhibit comparable Raman spectra. Subtle differences due exist, however, and quantitation may require the use of minor, rather than the most intense peaks (Shih et al., 2011). Hemicelluloses, due to their disorder and complexity, typically result in broader Raman bands than cellulose (Gierlinger and Schwanninger, 2006). Raman vibrational modes of cellulose are strongly affected by crystallinity and fiber orientation, enabling studies of cellulose polymorphs (Schenzel and Fischer, 2001). The dominant lignin vibrational mode near 1600 cm^−1^ is assigned to ring breathing, and therefore, is comprised of any phenyl-containing molecules, like flavonoids. If a biomass sample has a high extractable content, i.e., herbaceous feedstocks, the 1600 cm^−1^ peak will include contributions from lignin and other extractable molecules (Lupoi and Smith, 2012). Studies on lignin, therefore, require the efficient removal of extraneous species. Additionally, the 1600 cm^−1^ lignin peak contains overlapping signals from S, G, and H lignin monomers, complicating quantitative or semi-quantitative analyses between different biomass species (Lupoi and Smith, 2012). If the ratio of the monomers is known and does not significantly change between samples, and the samples have been exhaustively extracted, the 1600 cm^−1^ mode may be useful for evaluating relative lignin contents within feedstocks.

## Dispersive Raman Spectroscopy

**Figure 2:**
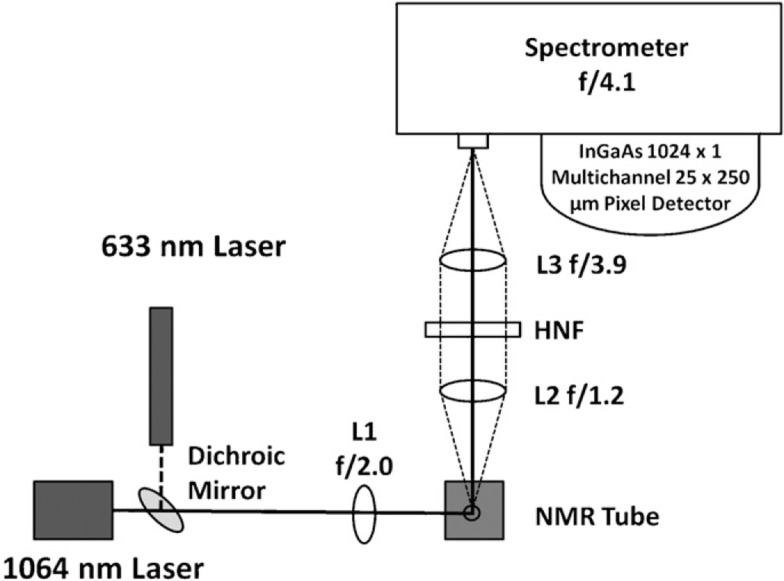
**Instrumental schematic of a 1064 nm dispersive multichannel Raman spectrometer**. The 1064 nm laser is focused onto a sample using a plano-convex lens (L1). The Raman scatter is collected with another plano-convex lens (L2) and focused onto the entrance slit of the spectrometer with a third plano-convex lens (L3). A holographic notch filter (HNF) is used to remove Rayleigh scattering. The spectrometer is equipped with a 1024-multichannel InGaAs detector. The helium–neon laser is oriented co-linearly with the 1064 nm laser, using a dichroic mirror, to facilitate instrumental alignment [reprinted with permission from Elsevier, Meyer et al. (2011)].

**Figure 3:**
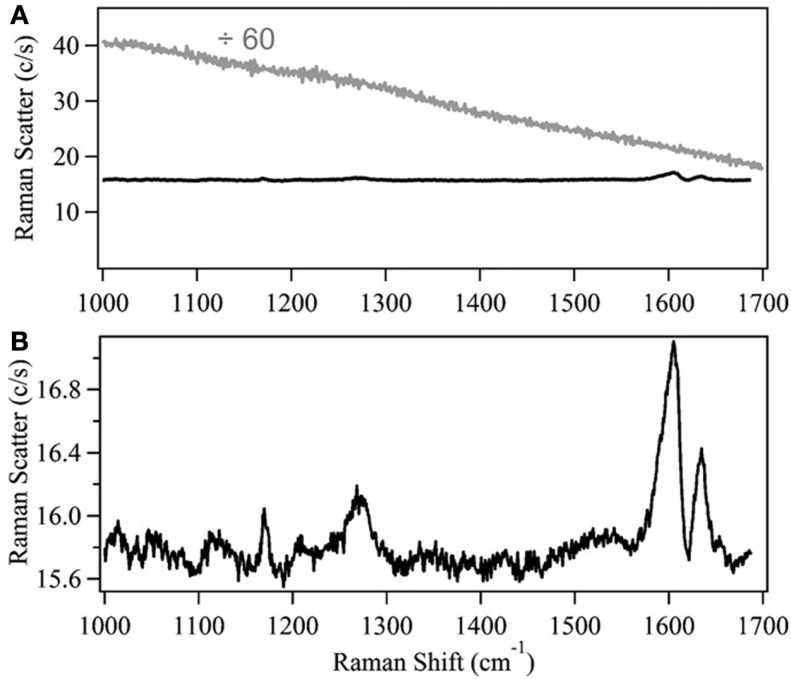
**Comparison of 785 and 1064 nm excitation wavelengths to evaluate lignin**. **(A)** Background-subtracted Raman spectra of 50 mg/mL lignin, dissolved in methanol, obtained using a dispersive 785 nm (gray) or 1064 nm (black) spectrometer. The 785 nm excitation spectrum has been divided by 60. **(B)** The 1064 nm excitation lignin spectrum, plotted on a smaller scale to elucidate spectral features [reprinted with permission from Elsevier, Meyer et al. (2011)].

As previously mentioned, NIR dispersive Raman spectroscopy can provide a suitable, less costly alternative to FT-Raman spectroscopy. Despite this, there are relatively few instances of researchers using this instrumental configuration (Roder and Sixta, 2005; Shih and Smith, 2009; Li et al., 2010, 2011, 2013; Meyer et al., 2011; Shih et al., 2011; Zakzeski et al., 2011; Lupoi and Smith, 2012; Ewanick et al., 2013; Gray et al., 2013; Azimvand, 2014; Iversen et al., 2014). A comparison between 785 and 1064 nm excitation sources revealed the latter to provide better signal-to-noise (S/N) when measuring hydrolytic lignin using home-built Raman spectrometers (Figure 2) (Meyer et al., 2011). The spectrum generated using the 785 nm laser exhibited a broad, featureless fluorescence background (Figure 3). The fluorescence emission peak maximum is expected to be in the visible region of the electromagnetic spectrum. When excited with the 785 nm light, however, a low intensity peak was detected that resembled the background measured in the Raman spectrum. Although the intensities of the peaks generated using the 1064 nm laser were relatively weak, the fluorescence was virtually eliminated (Figure 3). This instrumental configuration also provided higher S/N when compared to a commercial FT-Raman spectrometer using acquisition times greater than 15 s. The same system was used to develop a principal component regression (PCR) model to predict the S and G lignin content of a diverse assortment of feedstocks, including *Miscanthus*, switchgrass, poplar, and pine (Lupoi and Smith, 2012). The model was constructed from Raman spectral data conjoined with thioacidolysis/GCMS S and G lignin percentages.

**Figure 4:**
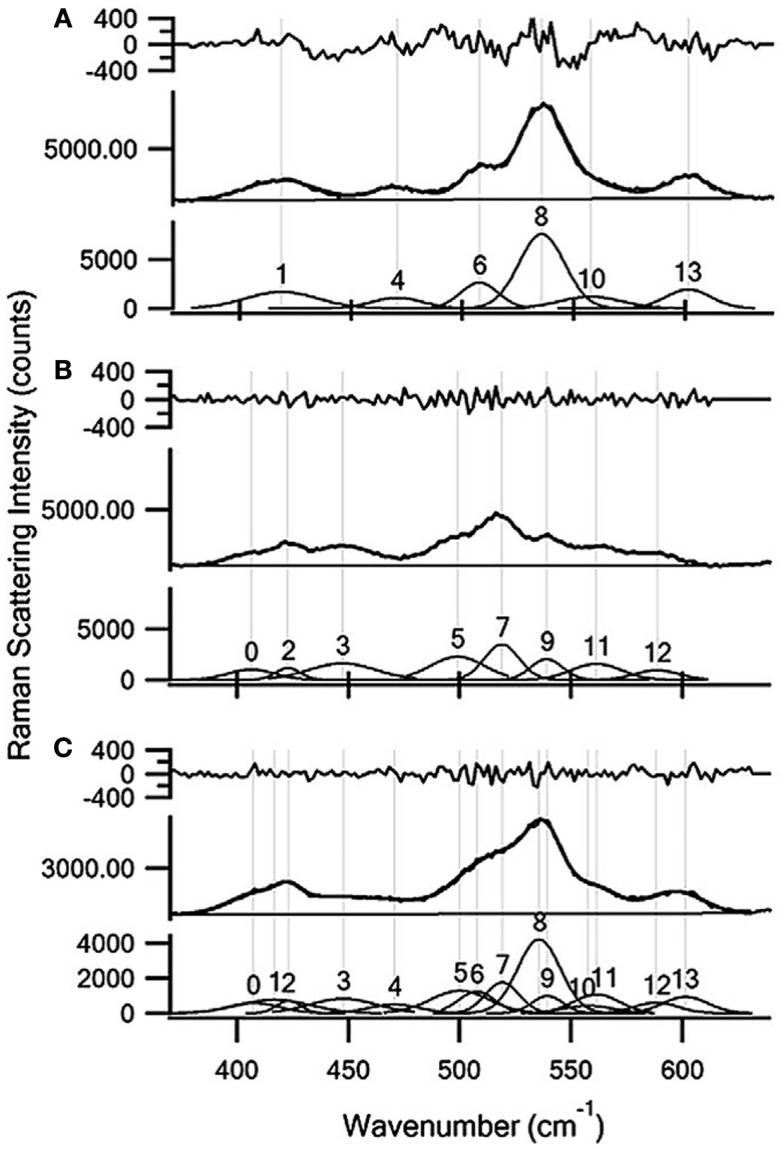
**Multi-peak curve fitting results for (A) 100 mg/mL xylose; (B) 120 mg/mL glucose; and (C) a 60 mg/mL glucose and 50 mg/mL xylose mixture in a soaking in aqueous ammonia hydrolysis broth**. The residual between the multi-peak fit and the experimental data is shown in the top panel. The residual is of the same order of magnitude as the spectral noise. The experimental spectra are shown in the middle panel (thicker line) and the fit results are shown in the bottom panel (thinner line) [reprinted with permission from Elsevier, Shih et al. (2011)].

The quantitation of glucose, xylose, and ethanol in complex matrices illustrated other novel applications of NIR, dispersive Raman spectroscopy (Shih and Smith, 2009; Shih et al., 2011). Raman methods were juxtaposed to those obtained using UV/visible (UV/VIS) spectrophotometry and headspace-GCMS. The UV/VIS methods required longer sample preparation and incubation steps. The GCMS analysis required the samples to be preheated to promote ethanol into the headspace, and had an experimental run time over 10 min per sample. The Raman measurements required relatively no sample preparation, and the spectral data was obtained using a 200 s acquisition time for glucose and xylose, and 100 s for ethanol. Another interesting feature of this work was the demonstration of the ability to simultaneously quantify glucose and xylose in hydrolyzate liquor using a multi-peak curve fit, with detection limits of 3 ± 2 and 1 ± 1 mg mL^−1^ for glucose and xylose respectively (Figure 4). The authors also evaluated the effects of various biomass pretreatment strategies on the ability to measure glucose. Soaking the biomass in aqueous ammonia or extracting using an aqueous ethanol solution resulted in lower detection limits. An acid pretreatment did not lower the detection limit, indicating that it was likely lignin and/or extractives like non-lignin phenolics that caused the higher spectral background, and thus elevated detection limits. These results clearly demonstrate the competence of Raman spectroscopy to measure hydrolysis and fermentation products rapidly and accurately.

**Figure 5:**
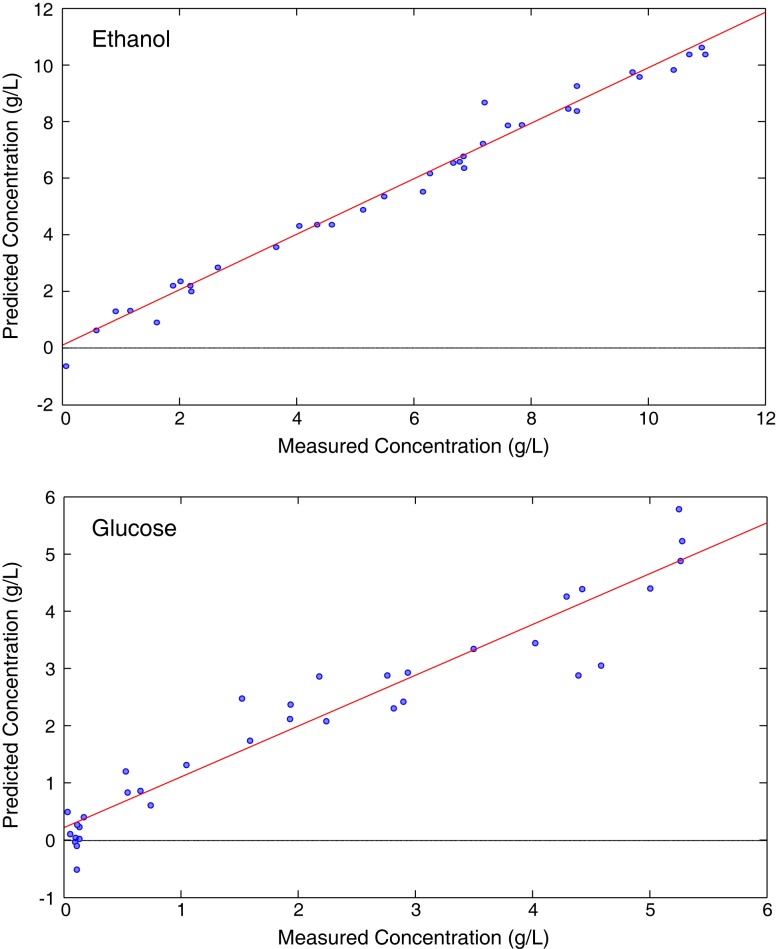
**Partial least squares models from the glucose fermentation comparing Raman spectral data with high-performance liquid chromatography [reprinted from an open access publication, with permission from BioMed Central, Ewanick et al. (2013)]**.

In addition to evaluating samples after the reaction has concluded (off-line), Raman spectroscopy can provide a valuable on-line, process monitoring tool, such as during the fermentation of glucose to ethanol. A fiber optic probe can be inserted directly into the reaction slurry. When glucose solutions were used as the starting feedstock, a partial least squares (PLS) model that married the Raman spectral data to standard HPLC ethanol and glucose measurements revealed correlation coefficients (*R*^2^) of 0.984 for ethanol and 0.92 for glucose (Figure 5) and good root mean standard errors of cross validation (RMSECV = 0.41, ethanol; 0.53, glucose) given the concentration range evaluated (Ewanick et al., 2013). When switchgrass hydrolyzate liquor was used as the fermentation feedstock, the measurement of glucose was significantly hindered. The hampered ability to quantify glucose resulted from its low concentration as well as the complex, heterogeneous nature of the hydrolyzate, which likely contained lignin-derived phenolics. The ability to measure the spectra of and use PLS to predict the concentration of ethanol was not impeded (*R*^2^ = 0.935, RMSECV = 0.60). A Raman spectrometer equipped with a 993 nm laser and a fiber optic probe enabled the real-time study of the formation of a complex assortment of products generated during a simultaneous saccharification and fermentation reaction (Gray et al., 2013). A simple univariate calibration using the 883 cm^−1^ vibrational mode allowed the quantitation of ethanol. The calibration was validated using a separate set of fermentation samples, and exhibited a *R*^2^ = 0.996, and a standard error of prediction (SEP) of 0.604. Multivariate PLS calibration models were generated for total starch, dextrins, maltotriose, maltose, glucose, and ethanol using HPLC standard measurements. The percentage error (defined as the SEP/modeling concentration range) was quite low for ethanol (2.1%), starch (2.5%), and dextrin (4.7%) when the calibration sets were broken up into low and high concentration series. The error was approximately two to seven times higher when only one calibration set was employed for these analytes. The percentage errors of glucose, maltose, and maltotriose were 12% or higher. On-line fermentation monitoring has been further illustrated using a similar instrumental configuration for the estimation of ethanol, glucose, and yeast concentrations (Iversen et al., 2014). Increasing concentrations of yeast were found to decrease the intensities of ethanol and glucose peaks caused by Mie scattering from the cells. The attenuation of the Raman signal was corrected using the 1627 cm^−1^ water band as an internal standard to adjust for the scattering from cell particulates. Once the spectra were corrected using the developed quadratic equations for each analyte, a simple linear regression allowed the quantitation of glucose and ethanol with high correlation (*R*^2^ = 0.99, ethanol; 0.995, glucose). This method also enabled the estimation of the yeast concentration.

As previously discussed, visible Raman excitation sources are not commonly employed, due to the intrinsic fluorescence of biomass; however, there have been some recent applications of visible Raman spectroscopy. A frequency doubled 1064 nm Nd:YAG green laser (532 nm for analysis) was used in an interesting study of laser-induced fluorescence (LIF) (Lähdetie et al., 2013). A variety of model compounds representing typical lignin sub-structures were evaluated, including erol, bioerol, dibenzodioxocin, 4-*O*-methylated bioerol, two synthesized phenolic compounds, and dehydrodivanillin-5-5′. Erol and dibenzodioxocin were easily measured with a relatively flat baseline. Bierol and 4-*O*-methylated bioerol revealed broad fluorescence backgrounds containing relatively no Raman bands. The synthesized molecules could be measured with only moderate spectral contributions from fluorescence; however, measurement of dehydrodivanillin-5-5′ resulted in the suppression of Raman modes by a fluorescence background. The authors conclude that the 5-5′ linkage is likely a strong source of LIF. Molecules that did not possess a conjugated link between two phenolic moieties did not exhibit fluorescence in the Raman spectra. Dibenzodioxocin, although it possesses the 5-5′ linkage, did not display a strong fluorescent background, which the authors deduce likely stems from the molecule’s rigid octagonal ring. Raman spectra from spruce wood and thermomechanical pulp (TMP), using the 532 nm laser, showed fluorescent backgrounds, however, the characteristic cellulose and lignin peaks were clearly discernible. When chemically treated pulps [kraft, enzymatic mild acidolysis lignin (EMAL), and milled wood lignin (MWL)] were analyzed, however, LIF was more pronounced. While EMAL and MWL isolation procedures are considered to be mild, retaining the native lignin structure, the analysis of these samples was distinctly different than lignin in wood. The authors hypothesized that the lack of a strong enough LIF background to prevent analysis of spruce likely arises from lignin being bound to the polymer matrix, preventing a release of fluorescence emission. Since EMAL and MWL are no longer connected to the polymeric network, a more malleable conformation results, which could trigger the increased fluorescence background. Other analyses using visible laser sources for Raman spectroscopy of biomass include the analysis of carbonaceous plant materials like bio-char (Ochoa et al., 2014; Tsaneva et al., 2014), and how changes in the cellulose crystallinity of delignified hybrid poplar samples affected the enzymatic hydrolysis yields (Laureano-Perez et al., 2006).

## Fourier-Transform Raman Spectroscopy

Fourier-transform Raman spectroscopy has been the most commonly used instrumental configuration for the analysis of biomass (Agarwal and Atalla, 1993; Sene et al., 1994; Agarwal and Ralph, 1997; Ona et al., 1997; Takayama et al., 1997; Kacurikova et al., 1998; Ona et al., 1998a; Ona et al., 1998b,c; Ona et al., 2000; Schenzel and Fischer, 2001; Sivakesava et al., 2001a; Sivakesava et al., 2001b; Kihara et al., 2002; Proniewicz et al., 2002; Agarwal et al., 2003; Ona et al., 2003; Cao et al., 2004; Vester et al., 2004; Agarwal and Kawai, 2005; Schenzel et al., 2005; Keown et al., 2007; Schulz and Baranska, 2007; Agarwal and Ralph, 2008; Keown et al., 2008; Schenzel et al., 2009; Agarwal and Atalla, 2010; Agarwal et al., 2010; Larsen and Barsberg, 2010; Agarwal, 2011; Agarwal et al., 2011; Chundawat et al., 2011; Larsen and Barsberg, 2011; Sun et al., 2012; Agarwal et al., 2013; Kim et al., 2013; Lupoi et al., 2014a; Wójciak et al., 2014; Lupoi et al., 2015). A recent study surveyed three high-throughput vibrational spectrometers (NIR, FTIR, and FT-Raman) to evaluate which was best suited for developing PLS models for predicting lignin S/G ratios (Lupoi et al., 2014a). Pyrolysis-molecular beam MS (pyMBMS) data from 245 diverse *Acacia* and eucalypt (*Eucalyptus* and *Corymbia*), encompassing 17 different biomass species, was coupled with NIR, FTIR, and Raman spectral data to build one global model. Iterations of different spectral processing techniques were conducted to see which permitted the most robust, accurate PLS model(s). The 245 samples were split into randomly generated 195-sample calibration and 50-sample validation sets. Additionally, the metrics used for evaluating each model were the result of three, independent, randomized models for each type of spectral transformation. The low error in the calibration and validation statistics indicated that these models were highly robust, as in most cases, it did not matter which samples were in the calibration or validation sets since every combination employed led to similar metrics. The best models (Table 2), based on RMSEP, were constructed using first-derivative, seven-point smoothed, Raman spectra with an extended multiplicative scatter correction (EMSC) (RMSEP = 0.13) and FTIR spectra that had been transformed using EMSC, first, and then the second derivative with 15-point smoothing (RMSEP = 0.13). In a follow-up study, the best Raman model was used to predict the lignin S/G ratio of 269 unknown *Acacia* and eucalypt samples (Lupoi et al., 2015). The calibration and validation sets used to generate the model were recombined to provide a larger data set, enabling more accurate predictions. The Raman predicted S/G ratios displayed no statistical differences from the pyMBMS measured results for all but one of the biomass species (Table 3). Additionally, the plant samples were ranked to illustrate which had the lowest and highest S/G ratios.

**Table 2:** **Comparison of partial least squares models using vibrational spectroscopy and pyrolysis-molecular beam mass spectrometry [reprinted from Lupoi et al. (2014a)]**.

Method	SEL validation^a^^,^^g^	SEP^b^^,^^g^	RMSEP^c^^,^^g^	r-Val^d^	*R*^2^ Val^e^	Outliers^f^
Raman 2nd deriv. (19pt) + SNV 32 scans	0.05	0.14	0.13	0.89 ± 0.04	0.79 ± 0.08	1
Raman 1st deriv. (7pt) + EMSC 32 scans	0.05	0.13	0.13	0.91 ± 0.02	0.83 ± 0.04	1
Raman EMSC + 2nd deriv. (15pt) 96 scans	0.05	0.14	0.15	0.90 ± 0.02	0.81 ± 0.04	0
Raman 2nd deriv. (15pt) + SNV 96 scans	0.06	0.17	0.16	0.86 ± 0.02	0.74 ± 0.04	0
MIR EMSC + 2nd deriv. (15pt)	0.05	0.14	0.13	0.87 ± 0.06	0.8 ± 0.1	1
MIR 2nd deriv. (17pt) + MSC	0.05	0.14	0.14	0.91 ± 0.01	0.83 ± 0.01	1
MIR 2nd deriv. (17pt) + SNV	0.05	0.15	0.15	0.87 ± 0.02	0.76 ± 0.03	1
NIR EMSC + 2nd deriv. (25pt)	0.06	0.19	0.20	0.79 ± 0.01	0.62 ± 0.01	0
NIR 2nd deriv. (25pt) + MSC	0.06	0.18	0.18	0.82 ± 0.04	0.67 ± 0.07	1
NIR 2nd deriv. (25pt) + SNV	0.06	0.22	0.21	0.80 ± 0.04	0.65 ± 0.07	1

*^a^Standard error of the laboratory for the validation data*.

*^b^Standard error of prediction*.

*^c^Root mean standard error prediction*.

*^d^Correlation coefficient for the validation set*.

*^e^Pearson coefficient of determination for validation*.

*^f^Number of outliers removed from validation models*.

*^g^Average errors of three randomly generated models using data provided. Models were not statistically different*.

*The numbers listed parenthetically reflect the degree of Savitzky–Golay spectral smoothing*.

*Statistical values are the average of three independent models*.

**Table 3:** **Comparison of pyrolysis-molecular beam mass spectrometry measured to Raman spectroscopy predicted syringyl/guaiacyl ratios^a^**.

Plant species	No. of samples	Reference range	pyMBMS S/G average	No. of samples	Prediction range	Raman S/G average	Predicted vs. reference comparisons (*p*-values)
*Acacia microbotrya*	5	1.2–1.5	1.3 ± 0.1	10	0.9–1.5	1.3 ± 0.2	0.83
*A. saligna*	4	1.4–1.9	1.7 ± 0.2	11	1.2–2.0	1.7 ± 0.2	0.69
*Corymbia citriodora* subsp. *citriodora*	17	2.1–2.8	2.4 ± 0.2	44	2.0–2.7	2.3 ± 0.1	0.61
*Corymbia* hybrids	47	1.6–2.8	2.2 ± 0.2	–	–	–	NA
*C. torelliana*	56	1.8–2.4	2.1 ± 0.1	–	–	–	NA
*C. citriodora* subsp. *variegata*	39	2.0–3.2	2.5 ± 0.3	61	2.2–2.7	2.5 ± 0.1	0.65
*Eucalyptus argophloia*	5	1.9–2.2	2.1 ± 0.1	5	1.7–2.0	1.8 ± 0.1	0.03
*E. cladocalyx*	3	2.3–2.6	2.5 ± 0.2	2	2.1, 2.4	2.2 ± 0.2	0.40
*E. cloeziana*	7	1.7–2.3	1.9 ± 0.2	15	1.7–2.4	2.1 ± 0.2	0.31
*E. crebra*	4	1.4–2.1	1.6 ± 0.4	6	1.2–2.1	1.8 ± 0.3	0.59
*E. dunnii*	4	2.2–2.8	2.5 ± 0.3	11	2.2–2.5	2.4 ± 0.1	0.36
*E. globulus*	11	2.3–3.0	2.6 ± 0.2	19	2.0–2.8	2.5 ± 0.2	0.15
*E. grandis*	2	1.9, 2.2	2.0 ± 0.2	13	1.9–2.4	2.2 ± 0.1	0.23
*E. kochii*	5	1.9–2.3	2.2 ± 0.2	10	1.7–2.5	2.2 ± 0.2	0.76
*E. longirostrata*	8	2.1–2.4	2.2 ± 0.1	7	2.0–2.3	2.2 ± 0.1	0.86
*E. loxophleba*	7	2.1–2.6	2.4 ± 0.1	23	2.2–2.7	2.4 ± 0.1	0.22
*E. moluccana*	5	2.0–2.5	2.2 ± 0.2	11	1.8–2.5	2.2 ± 0.2	0.91
*E. occidentalis*	6	2.1–2.5	2.4 ± 0.2	9	2.2–2.6	2.4 ± 0.1	0.55
*E. polybractea*	8	2.0–2.7	2.3 ± 0.2	12	1.9–2.5	2.2 ± 0.1	0.40

*^a^Data compiled from Lupoi et al. (2014a), “High-throughput prediction of eucalypt lignin syringyl/guaiacyl content using multivariate analysis: a comparison between mid-infrared, near-infrared, and Raman spectroscopies for model development,” *Biotechnology for Biofuels*, Volume 7, p. 93 and Lupoi et al. (2015), “High-throughput prediction of *Acacia* and eucalypt lignin syringyl/guaiacyl content using FT-Raman spectroscopy and partial least squares modeling” *Bioenergy Research*, open access*.

Lignin S/G ratios of Eucalyptus, sorghum, switchgrass, maize, and *Arabidopsis* were evaluated using the deconvolution of FT-Raman spectra into peaks identified as representative of S or G lignin monomers (Sun et al., 2012). The specific vibrational modes unique to the different biomass constituents were determined through the measurement of cellulose, xylan, and various model compounds, such as coniferaldehyde, sinapic acid, creosol, 5-methylpyrogallol trimethyl ether, sinapinaldehyde, and sinapyl alcohol. Spectrally resolved peaks corresponding to S or G lignin derivatives were then applied to the biomass samples. The ratios of the resolved S and G peaks were determined and compared to pyGCMS results. The ratios calculated using Raman spectroscopy were consistently higher than those measured using pyGCMS, which could be due to the presence of polysaccharide vibrational modes overlapping with spectral regions designated for each monomer. The deconvolution process itself also contributed to some false peaks such as an artificial S band for pine, a plant known to contain no real S components. Nonetheless, a calibration curve generated using the pyGCMS and Raman calculated ratios resulted in a reasonable correlation (*R*^2^ = 0.983). *Arabidopsis* mutants were used to validate the regression model, resulting in a better correlation with the pyGCMS S/G ratios.

When analyzing lignocellulosic materials with Raman spectroscopy, a phenomenon termed “self-absorption” must be considered (Agarwal and Kawai, 2005). Self-absorption occurs when scattered photons are re-absorbed back into the analyte, resulting in an attenuation of the scattered light reaching the detector. This can be visually identified in a Raman spectrum by the decrease in intensity of a vibrational mode where the molecule absorbs light. An analysis of cellulose filter paper, spruce TMP, and MWL illustrated that most of the spectral suppression occurred at the 2895 cm^−1^ C–H peak of the filter paper and TMP (Agarwal and Kawai, 2005). Evaluation of the spectra pointed to cellulose and water as the main contributors of self-absorption, while lignin’s involvement was unmeasured. FT-Raman spectroscopy enabled the analysis of the structure of MWLs produced from hard- and softwoods and chemically treated black spruce (Agarwal et al., 2011). The Raman spectra revealed distinct changes when differentiating the untreated to pretreated samples. Acetylation and methylation produced sizeable changes in aliphatic C–H vibrational modes, and also resulted in the formation of several new peaks.

The viability of FT-Raman spectroscopy for monitoring a bioethanol process has also been explored (Sivakesava et al., 2001b). Glucose, ethanol, and optical cell density were evaluated during ethanol fermentation. Raman spectra were coupled with HPLC results for the construction of PLS and PCR models. Although the predictions of glucose and ethanol were acceptable, the cell density modeling proved to be more erroneous due to the weak scattering generated from the cultures. Another study analyzed glucose, lactic acid, and cell density, at-line, during a lactic acid fermentation process (Sivakesava et al., 2001a). PLS models generated using IR, NIR, and Raman spectral data were contrasted, with the Raman models having the second lowest SEP in glucose prediction. The Raman SEP of lactic acid and cell density predictions ranked third between the three instruments. The authors attribute this lack of accuracy to the fact that glucose, lactic acid, and proteins have weaker Raman signals compared to IR spectroscopy.

## Resonance Raman Spectroscopy

Resonance Raman (RR) spectroscopy is achieved when a molecule has an electronic absorption that overlaps with the excitation source wavelength, resulting in the promotion of the molecule to a real, rather than a virtual, electronic state. In complex analytes such as biomass, molecules resonating with the excitation source will be selectively enhanced. For example, lignin has an electronic absorption in the UV region, leading to increased lignin spectral intensities when UV lasers are employed. This resonance allows lignin to be preferentially studied while polysaccharides generate limited spectral response. An advantage of evaluating lignin with UVRR, as previously discussed, is that the lignin can be measured *in situ*. This allows a more pragmatic analysis of lignin structure, since the techniques commonly employed to extract or isolate lignin from plants are known to alter the lignin. Another benefit to using UV lasers is that fluorescence, ubiquitous in visible Raman spectroscopy, and still a hindrance at some shorter NIR wavelengths, is not problematic. The analysis of lignin model compounds using UVRR enabled S, G, and H lignin markers to be characterized (Saariaho et al., 2003, 2005). The use of a tunable argon laser allowed three different excitation wavelengths to be evaluated: 229, 244, and 257 nm (Saariaho et al., 2003). The lignin S, G, and H markers were preferentially enhanced based upon which excitation wavelength was used. H lignin structures showed the strongest enhancement when 244 nm was employed, while G moieties were more intense when 257 nm was used. The spectra generated from S functionalities were essentially indistinguishable when using either 244 or 257 nm. A follow-up study utilized PLS to determine the specific wavelengths correspondent to each type of lignin monomer, as well as condensed structures, conjugated C = C and C = O, and stilbenes (Saariaho et al., 2005). The authors note that using multivariate analysis in this fashion can aid in qualitatively interpreting complex spectra of polymeric lignin, since the UVRR spectra typically have broad peaks. The evaluation of the PLS model loadings plots allowed the identification of important vibrational modes corresponding to the different model compound classes.

The functional groups of lignin contribute to its chemical properties and its valorization potential. Phenolic moieties, one of the principal functionalities in lignin, define the reactivity and solubilization of lignin (Zakis, 1994). The ionization of phenolic species in alkaline media results in a concomitant shift in the vibrational modes of lignin in pulps and lignin model compounds (Warsta et al., 2012). Shifts from 8 to 35 cm^−1^ were measured when the pH was increased from 6 to 12. In general, as the pH became more alkaline, a shift to lower wavenumbers was detected. When wood pulps were analyzed, a less pronounced shift resulted, since the pulps have less phenolic functionalities than model compounds. When non-phenolic 3, 4-dimethoxytoluene was measured, no shift was detected, indicative that the shifting occurred due to ionization of the phenolic group. Increases in pH also resulted in augmented band intensities; however, the band intensity was still directly proportional to analyte concentration, as exemplified by the construction of a calibration curve for guaiacol. While these bands were detected at more neutral pH levels, the enhancement of these bands at strongly basic pH provided a more detailed structural analysis. The authors suggest that the shifting of the aromatic band near 1600 cm^−1^ from increasing the alkalinity of the matrix may aid in determining the amount of free phenolic groups (for example, an 11 cm^−1^ shift can be expected if *all* of the phenylpropanoid functionalities have a free phenolic group).

Ultraviolet resonance Raman enabled the analysis of extractable lipophilic and hydrophilic components from Scots pine wood resin (Nuopponen et al., 2004b,c). The authors employed a tunable argon laser set to one of three different excitation wavelengths: 229, 244, or 257 nm. The level of the enhancement for different structures depended on the particular laser wavelength employed. Molecules such as resin (dehydroabietic, abietic, and pimaric type) and fatty acids, sitosterol, and sitosterol acetate were evaluated as standards, in hexane extracts from the biomass, and in solid wood samples. Double-bond moieties, such as those found in alkenes, were resonantly enhanced using the UV laser wavelengths. When the 257 nm wavelength was used, compounds with isolated double bonds provided the most information, while the 229 nm wavelength was more useful for analyzing conjugated resin acids. Additionally, the 257 nm laser was best suited for studying sapwood hexane extracts, while either the 229 or the 244 nm lasers could be employed for evaluating heartwood extracts. The measurement of solid wood revealed a vibrational mode at 1650 cm^−1^, indicative of unsaturated wood resin constituents. For the hydrophilic extractables, only the 244 and 257 nm wavelengths were used. Aromatic and unsaturated moieties of pinosylvin and chrysin were found to be resonantly enhanced. Wavelength selection had a minimal effect on chrysin analysis. The heartwood acetone/water extract included pinosylvin plus resin and fatty acid markers. The sapwood extract contained oleophilic structures of the resin and fatty acids, as well as some guaiacyl modes. The measurement of Scots pine knotwood unveiled an abundant resin contribution, illustrating that the resin was more resonantly enhanced than lignin. These two analyses have demonstrated the competence of UVRR to selectively analyze extractable compounds. Although extractives are a smaller proportion of biomass compared to polysaccharides and lignin, they have significant impacts on plant properties and may also present a source of bio-based chemicals.

A UVRR method was established for quantifying lignin in bleached hardwood kraft pulps (Jaaskelaeinen et al., 2005). Lignin quantification techniques typically are developed using unbleached biomass, and therefore are not readily transferable to bleached samples. A strong linear correlation (*R*^2^ = 0.987) was calculated when the 1604 cm^−1^ peak was normalized to the 1093 cm^−1^ cellulose peak, and plotted against increasing lignin concentration. A 244 nm excitation wavelength provided more accurate lignin content measurements, since the use of 257 nm resulted in more fluctuations in the spectral baseline. The Raman measured lignin contents were compared with kappa numbers measured using a standard method, and were found to linearly correlate. Other applications of UVRR include the degradation of lignin following a chemical treatment such as bleaching (Halttunen et al., 2001; Mononen et al., 2005; Jaaskelainen et al., 2006; Läehdetie et al., 2009) or steam treatment (Nuopponen et al., 2004a), the changes in TMP after laccase treatments (Lähdetie et al., 2009), photodegradation using an UV laser (Pandey and Vuorinen, 2008), and an evaluation of 25 diverse tropical hardwoods using UVRR spectral data and principal component analysis (PCA) (Nuopponen et al., 2006).

Resonance Raman spectroscopy using visible excitation sources with Kerr-gated fluorescence rejection has enabled structural analyses of lignin that were previously unattainable (Barsberg et al., 2005, 2006). A Kerr-gate is a device consisting of two polarizers and a Kerr medium (carbon disulfide, in this instrument). When closed, the polarizers blocked scattered photons from reaching the detector. The Kerr-gate provided a time-window of 4 ps to collect Raman spectra free from fluorescence, a phenomenon occurring on a nanosecond timeframe. Once the Raman data had been acquired, the Kerr-gate was switched to the closed position, thereby blocking fluorescence. Syringyl moieties were resonantly enhanced when a 400 nm laser was used, whereas the use of 500 nm light caused a reduction in selectivity. The effects of laccase plus various mediators on beech lignin were studied using both excitation wavelengths and RR difference spectra. In a follow-up study, the authors successfully measured lignin radicals produced enzymatically using laccase (Barsberg et al., 2006). A 1570 cm^−1^ band was measured in dry wood, regardless of the type of biomass was analyzed. When wet beech was evaluated, a lignin radical peak at 1606 cm^−1^ was detected. Density functional theory was used to correlate the experimental results with the predicted vibrational modes of lignin radicals, and indicated that the radicals were formed from syringyl and guaiacyl moieties in beech and spruce, respectively. RR difference spectra were calculated to subtract spectral contributions from the main lignin peak near 1600 cm^−1^. The radicals could only be detected when the 500 nm light was used to generate Raman scatter.

Resonance Raman spectroscopy coupled with Kerr-gated fluorescence suppression allowed the measurement of strongly fluorescent chemical pulps using 400 nm light (Saariaho et al., 2004). Although these pulps are not typically assessable, due to lignin fluorescence, the use of the Kerr-gate permitted a 250-fold reduction in the fluorescence background, enabling much weaker Raman bands to be detected. Chromophoric vibrational modes at 1605 and 1655 cm^−1^ were measured in peroxide-bleached pulps, while only the 1605 cm^−1^ was identified in biomass treated with chlorine dioxide. When a 257 nm laser was used to evaluate the pulps, the intensity of the aromatic lignin peak was approximately 20-times higher than the main cellulose mode. The square root of the ratio of the 1605 cm^−1^ vibrational mode to the 1098 cm^−1^ peak correlated linearly with brightness percentage, as measured using a standard method. The authors concluded that while UV excitation preferentially probed lignin, visible lasers allowed the detection of chromophoric lignin structures. Lignin remaining in chemically treated pulps could be quantified using RR spectroscopy, although the detection limit can be lowered when UV lasers are employed.

## Raman Imaging

Raman imaging techniques have enabled the visual examination of biomass cell and cell wall structure, and the evaluation of real-time changes in the morphology and chemical content of plants, such as after different pretreatment strategies. These experiments have provided a glimpse into the chemistry of plants before and after treatments, permitting researchers to identify the biomass modification approaches best suited for reducing recalcitrance and increasing yields from downstream conversion into simple sugars. The laser can be focused to small spot sizes, enabling minute areas of interest to be evaluated. Instrumental advances have allowed the rapid acquisition of images with short integration times, preventing the photodegradation of the sample. Another advantage of Raman imaging, juxtaposed to other imaging techniques, is that no staining or embedding of the sample is required. Raman spectra are collected from the sample, as the instrument passages the sample to defined locations using a set step-size, resulting in a plethora of structural and chemical data that can be daunting to analyze. Multivariate analysis, coupled with imaging techniques, has enabled enhanced data mining for valuable information.

**Figure 6:**
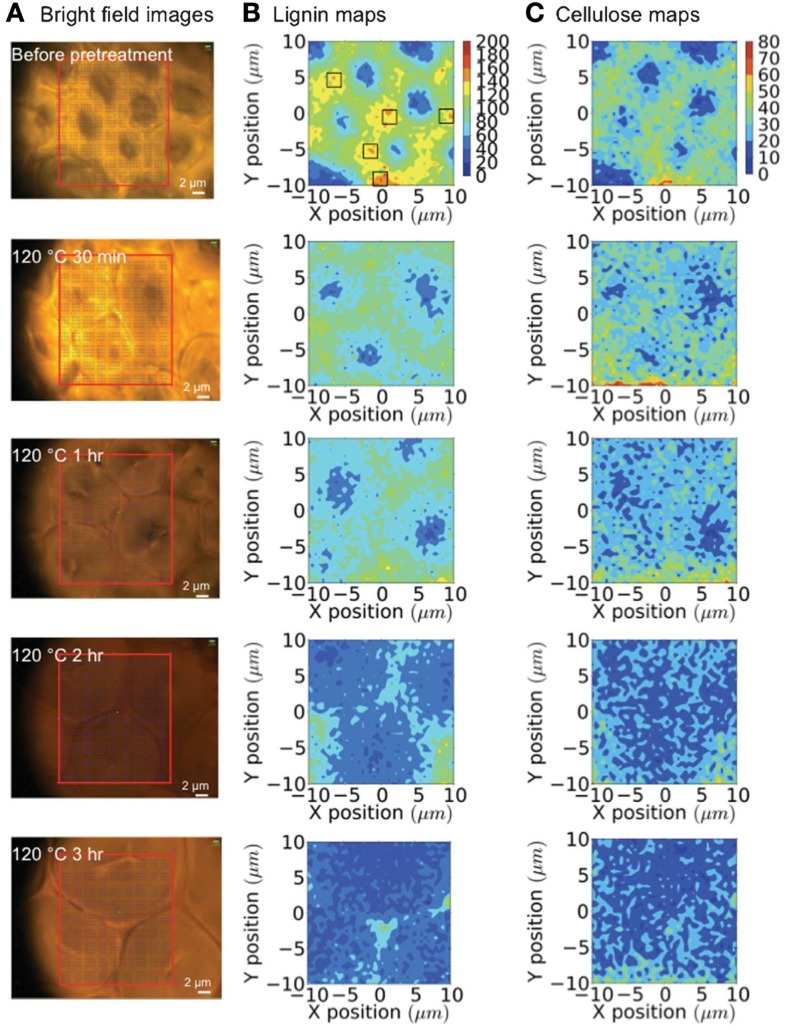
**Raman mapping of tracheids during an ionic liquid pretreatment**. **(A)** Bright-field microscopy images; **(B)** lignin maps (black boxes are the locations of cell corners); and **(C)** cellulose maps generated over 0–3 h of pretreatment [reprinted with permission from the Royal Society of Chemistry, Sun et al. (2013)].

**Figure 7:**
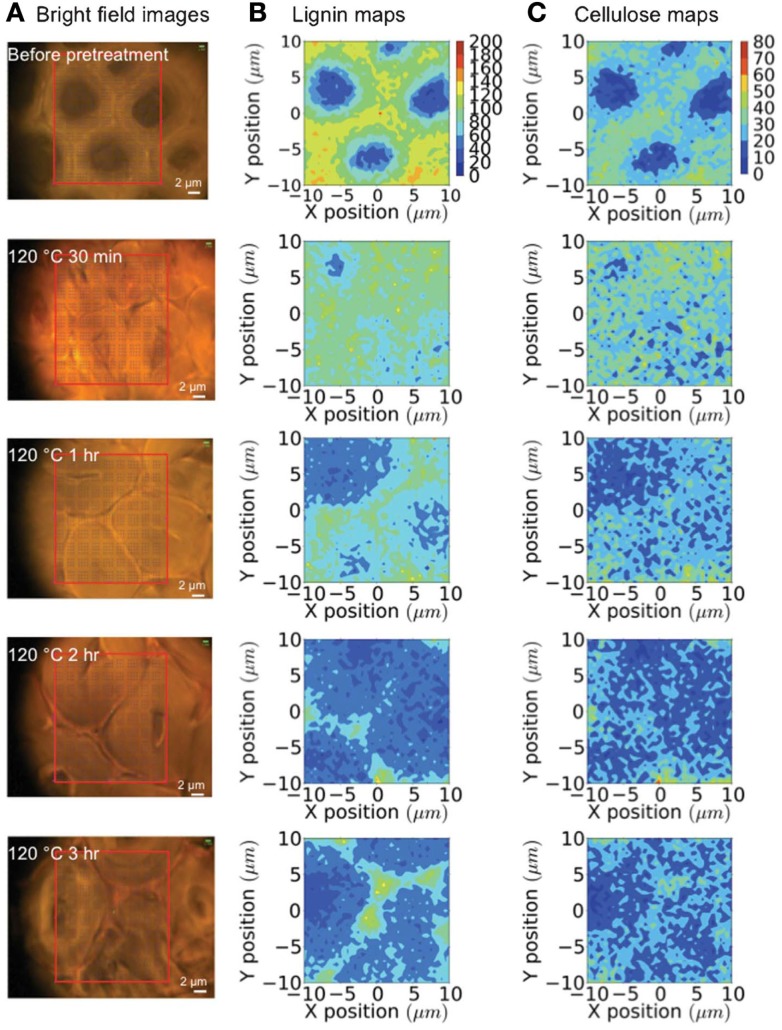
**Raman mapping of sclerenchyma cells during an ionic liquid pretreatment**. **(A)** Bright-field microscopy images; **(B)** lignin maps (black boxes are the locations of cell corners); and **(C)** cellulose maps generated over 0–3 h of pretreatment [reprinted with permission from the Royal Society of Chemistry, Sun et al. (2013)].

Raman microspectroscopy has been used to evaluate how a room temperature pretreatment with the ionic liquid (IL) 1-*n*-ethyl-3-methylimidazolium acetate modified the cell walls of poplar (Lucas et al., 2011). A 785 nm diode laser was used to collect spectral data from 50 μm poplar sections. Raman spectra from untreated poplar revealed the characteristic vibrational modes from cellulose, hemicellulose, and lignin. When the wood was swollen with water, the same peaks were identified; however, the intensities differed from the untreated samples. The intensity ratio of the 1460 cm^−1^ cellulose peak to the 1605 cm^−1^ lignin peak decreased, which signified diminutions in the cellulose-abundant S2 sub-layer compared to the hydrophobic, lignin-rich compound middle lamella (CML) region. The authors conclude that the swelling must be pushing the fibers apart, and progressing into more amorphous cellulose regions since crystalline cellulose fibers are recalcitrant to water penetration. The Raman spectra of the IL treated poplar samples depicted strong signals from the IL itself. When the samples were washed with water prior to analysis, the spectra showed no traces of IL vibrational modes, and resembled the water-swollen poplar Raman spectrum, leading to the conclusion that both the water and IL treatments led to similar overall cell wall compositions. Confocal Raman spectroscopy using a 785 nm diode laser enabled an evaluation of tissue-specific changes when pretreating corn stover with the IL 1-ethyl-3-methylimidazolium acetate (Sun et al., 2013). A temporal study was conducted to gage the lignin and cellulose remaining in the plant cell walls during the IL pretreatment at 120°C using 0, 30 min, 1, 2, and 3-h time points. To assess the changes brought about by the IL treatment, tracheids, sclerenchyma, and parenchyma cell structures were probed (see Figures 6 and 7). Before pretreating the corn stover, cellulose and lignin concentrations were highest in the cell corners (CCs) and CML portions of the three cell structures and in the secondary walls of the sclerenchyma and parenchyma cell types. The lignin content was measured to decrease rapidly during the IL treatment, while no preferential cellulose dissolution was detected. The IL pretreatment is known to cause swelling of the secondary wall, but not of the CML. Accordingly, more significant swelling was observed in tracheid and sclerenchyma cells than parenchyma cells, which are composed of primary cell walls. Although tracheids contained higher lignin concentrations and thicker walls than parenchyma cells, the lignin dissolution occurred more rapidly in the tracheid cells. Confocal Raman microscopy was also employed to evaluate normal and tension wood sections from poplar (Gierlinger and Schwanninger, 2006). The allocation of cell wall components was calculated following the integration of distinct vibrational modes. The Raman images of normal wood illustrated higher lignin concentrations in the CCs and the CML, and increased cellulose content in the S2 layer of parenchyma ray cells and two lesser layers located on each side of the CML, presumed to be S1. A higher fluorescence background was measured for CCs and the CML, which is expected due to the greater lignin concentrations in these regions. Analysis of tension wood samples revealed lignin to be localized in CCs and the CML, while no lignin was detected in the gelatinous, or G-layer. Signals from lignin increased, however, in the lumen. Aromatic compounds were measured to coalesce along an inner region of the G-layer, and were also found deeper in the G-layer, toward the CCs of the S2 layer.

Many applications of Raman imaging utilize NIR excitation sources. Visible excitation, however, has been demonstrated as offering a higher energy source for obtaining Raman images. A novel, polarized 633 nm laser was used to attain images of black spruce cross-sections (Agarwal, 2006). Fluorescence was efficiently blocked by acquiring data in confocal mode using a 100 μm pinhole. Lignin concentrations were highest in the CCs, concurrent with other studies, but were not profoundly different in the CML and secondary wall. Coniferaldehyde and conifer alcohol distribution, using the 1650 cm^−1^, was measured to correspond with lignin. Cellulose localization followed an opposite pattern to lignin distribution (high S2 and low CC and CML concentrations). A confocal Raman microscope, equipped with a 532 nm laser and a 100 μm pinhole, was used to characterize black cottonwood (Perera et al., 2011). Given the heterogeneity of the sample, the abundance of spectral information, and spectrally unresolved vibrational modes, the authors developed a new analysis strategy to aid in determining the structural characteristics and chemical composition of the wood. The method encompasses three main phases: spectral preprocessing, stepwise clustering, and estimation of spectral profiles of pure components and their respective weights. The spectral processing included wavelet analysis to remove noise, second-derivative transformations to remove contributions from fluorescence, and PCA to reduce the amount of variables as well as reduce noise from the data matrix. Stepwise clustering was achieved using *k*-means clustering to classify the samples according to a preordained number of groups. The image can then be reconstructed using the cluster groupings, facilitating the identification of diverse sub-layers within the cell wall. The last step involves determining which factors are important in contributing to the distinct localization of different cell wall components in the images. A technique called spectral entropy minimization methodology allowed the pure components spectra to be captured. Estimated pure polysaccharide and lignin spectra were generated. Pure cellulose and hemicellulose components could not be generated due to the structural, and therefore, spectral resemblance between the two polysaccharides. The lignin spectrum included regions typically assigned to lignin monomers, permitting an *in situ* analysis of monolignol composition. The authors note that this is not possible with routine data processing techniques, since the lignin monomers have significant spectral overlap with carbohydrate vibrational modes. The partitioning of lignin and carbohydrates in the images was determined by subtracting first the pure lignin and then the pure carbohydrate spectra from the average spectra determined for each cluster. The image analysis procedure can be extended to other types of spectral data such as IR, MS, or fluorescence. In a follow-up study, this method was employed to evaluate the S and G lignin contents of *Arabidopsis*, *Miscanthus*, and poplar. Spectral distinctions between the three plants were clearly discernible in the estimated lignin spectra, indicative of differences in S, G, and H contents. The *Miscanthus* spectrum was less intricate than the dicots, which, the authors deduce is illustrative of *Miscanthus* having a higher percentage of non-condensed lignin. Lignin S/G ratios were calculated to be 0.5 ± 0.08, 0.6 ± 0.1, and 1.9 ± 0.2 for *Miscanthus*, *Arabidopsis*, and poplar, respectively. The S/G ratios within different cell wall structures could also be calculated (0.8 ± 0.1 for *Miscanthus* xylary fiber cells, 0.6 ± 0.1 for *Miscanthus* interfascicular cells of basal stems). A transgenic poplar sample, in which the monolignol biosynthesis gene encoding for 4-coumarate-CoA ligase was suppressed, revealed reduced total lignin contents and decreased S/G ratios. These examples demonstrate the power of Raman spectroscopy coupled with chemometric techniques to exhaustively extract obscured information from the spectra.

Coherent anti-Stokes Raman scattering microscopy can be used to obtain images of biomass devoid of fluorescence (Zeng et al., 2012; Pohling et al., 2014). In CARS, multiple lasers interact with the analytes, termed pump, probe, or Stokes excitation sources. These lasers are used to generate the anti-Stokes photons. When the frequency difference between the pump and Stokes lasers is tuned to coincide with a specific Raman vibrational mode, the signal is enhanced. CARS intensities are stronger than those obtained using spontaneous Raman spectroscopy, leading to increased sensitivity, and shorter acquisition times. Spectral assignments in CARS spectra are identical to those assignments made using traditional Raman spectra. Wood samples of birch, oak, and spruce were evaluated using CARS microscopy (Pohling et al., 2014). Standards of pure cellulose, xylan, and lignin were measured to establish indicative marker bands. Through the use of spectrally broad lasers, the CARS protocol can probe multiple vibrations (MCARS). Using a technique called the maximum entropy method, Raman spectra could be extracted from the MCARS spectral data, revealing spectra that resembled standard spectra collected with spontaneous Raman spectroscopy, minus fluorescence contributions. Transverse and longitudinally oriented cuts of the wood samples illustrated the cell wall structure and composition. Cellulose, hemicellulose, and lignin were localized allowing the assignment of cellulose-rich secondary walls, and lignin-rich intercellular space. The longitudinally cut images showed polarization dependence. Stronger cellulose signals were detected using horizontal polarization, while more intense lignin peaks were measured using vertical polarization. A semi-quantitative assessment of cellulose, hemicellulose, lignin, and water illustrated the significance of polarization in the longitudinal plant sections, as there was greater disparity in the results when measured with horizontal or vertical polarization.

Coherent anti-Stokes Raman scattering, however, experiences an electronic background that can alter spectral data, obscuring the quantitation of analytes from CARS imaging techniques (Li et al., 2005). SRS microscopy provides orders-of-magnitude higher spectral signals, eliminating the effects of the higher background (Freudiger et al., 2008). The SRS phenomenon is similar to that observed in CARS. Two lasers are overlapped and focused onto the analyte. When the difference frequency of the two lasers resonates with a vibrational mode in the sample, the rate at which photons migrate to higher vibrational levels is enhanced due to stimulated photon excitation. Energy transfers only occur when in resonance with a molecule’s fundamental vibrational mode(s). Although the signals are weak and obscured in the background produced from the laser, the laser noise can be eliminated by using a high-frequency (>1 MHz) amplitude modulation/lock-in detection procedure (Saar et al., 2010). Like CARS, the assignments of vibrational modes are equivalent to those generated from spontaneous Raman scattering. Also analogous to spontaneous Raman spectroscopy, signals produced using SRS are linearly dependent on analyte concentration. SRS microscopy was employed to evaluate the real-time processing of corn stover (Saar et al., 2010). The images were acquired in approximately 3 s, whereas the same image would have required nearly 2 h using spontaneous Raman scattering. Cellulose and lignin localization were generated in the images, without using labels or staining, by tuning the frequency difference of the two lasers to the well-known vibrational modes of each biopolymer. The validity of this technique was confirmed by comparing with common staining techniques, such as phloroglucinol for lignin detection. The vessel, tracheid, and fiber cells revealed significant lignification compared to phloem cells. The cellulose content was more uniformly dispersed throughout the cells, juxtaposed to lignin. Areas of higher and lower cellulose and lignin concentration could be detected, an observation that is more challenging in CARS, due to the inability to separate the signal from the higher background. The authors used this method to monitor the delignification of corn stover using a sodium chlorite treatment. An eightfold decrease in lignin content was measured while the cellulose content remained relatively unchanged. Analysis of the SRS images provided information on where lignin was preferentially removed from the corn stover during the bleaching process. The bleaching rates were fastest for the lignin contained in the phloem and CCs. Parenchyma, tracheid, vessel, and fiber cells demonstrated similar delignification patterns, signifying similar accessibilities of lignin to sodium chlorite.

Other Raman imaging applications include an analysis of the structural changes in polyaromatic molecules and non-aromatic moieties following the carbonization of Japanese cedar, cotton cellulose, and lignin at 500–1000°C (Ishimaru et al., 2007), studies on deformation properties of native and regenerated celluloses (Hamad, 2008), the monitoring of structural and chemical changes in *Miscanthus x giganteus* following a sodium hydroxide treatment (Chu et al., 2010), the localization of cellulose and lignin in corn stover and *Eucalyptus globulus* (Sun et al., 2010), the *in situ* detection of a single carotenoid crystal (Baranska et al., 2011), and the characterization of cellulose nanocrystal (CNC)-polypropylene composites, and determine the spatial distribution of the CNC in the filaments (Agarwal et al., 2012).

## Conclusion

As the search for ideal wild-type or transgenic biofuel and biomaterial feedstocks progresses, methods that rapidly and accurately screen large arrays of different plants are becoming essential. Raman spectroscopy, in its diverse configurations, has proven to be a viable asset to these qualitative and quantitative studies. As instrumental innovations evolve, such as field-portable devices, measurements of the feedstocks can be conducted in their natural environments, reducing the need for time-consuming sampling protocols. The construction of robust, multivariate predictive models coupled to Raman spectral data will increase experimental throughput, thereby narrowing the pool of potential plants suitable for downstream renewable energy applications. Raman imaging techniques have empowered researchers to evaluate deconstruction strategies in real-time, providing both fundamental insights into how specific reagents affect the morphology of the biomass, and also the ability to nominate or exclude the pretreatment method based on the efficiency of rendering the cell wall-less recalcitrant based on end-product yields. The extent of endeavors explored for the characterization of lignocellulosic biomass using Raman spectroscopy continues to escalate. Future advancements in instrumentation, multivariate analysis modeling, and the revolutionary ways in which Raman spectroscopy is utilized will continue to proffer researchers a versatile, non-destructive, non-invasive, user-friendly, high-throughput analytical tool.

## Conflict of Interest Statement

The authors declare that the research was conducted in the absence of any commercial or financial relationships that could be construed as a potential conflict of interest.
